# Differential Expression of Proteins Associated with Bipolar Disorder as Identified Using the PeptideShaker Software

**DOI:** 10.3390/ijms242015250

**Published:** 2023-10-17

**Authors:** Alexander A. Seregin, Liudmila P. Smirnova, Elena M. Dmitrieva, Maria G. Zavialova, German G. Simutkin, Svetlana A. Ivanova

**Affiliations:** 1 Mental Health Research Institute, Tomsk National Research Medical Center, Russian Academy of Sciences, Tomsk 634014, Russia; apocalips1991@gmail.com (A.A.S.);; 2 Skolkovo Institute of Science and Technology, Moscow 121205, Russia

**Keywords:** bipolar disorder, proteome, PeptideShaker, mass spectrometry, serum

## Abstract

The prevalence of bipolar disorder (BD) in modern society is growing rapidly, but due to the lack of paraclinical criteria, its differential diagnosis with other mental disorders is somewhat challenging. In this regard, the relevance of proteomic studies is increasing due to the development of methods for processing large data arrays; this contributes to the discovery of protein patterns of pathological processes and the creation of new methods of diagnosis and treatment. It seems promising to search for proteins involved in the pathogenesis of BD in an easily accessible material—blood serum. Sera from BD patients and healthy individuals were purified via affinity chromatography to isolate 14 major proteins and separated using 1D SDS-PAGE. After trypsinolysis, the proteins in the samples were identified via HPLC/mass spectrometry. Mass spectrometric data were processed using the OMSSA and X!Tandem search algorithms using the UniProtKB database, and the results were analyzed using PeptideShaker. Differences in proteomes were assessed via an unlabeled NSAF-based analysis using a two-tailed Bonferroni-adjusted *t*-test. When comparing the blood serum proteomes of BD patients and healthy individuals, 10 proteins showed significant differences in NSAF values. Of these, four proteins were predominantly present in BD patients with the maximum NSAF value: 14-3-3 protein zeta/delta; ectonucleoside triphosphate diphosphohydrolase 7; transforming growth factor-beta-induced protein ig-h3; and B-cell CLL/lymphoma 9 protein. Further exploration of the role of these proteins in BD is warranted; conducting such studies will help develop new paraclinical criteria and discover new targets for BD drug therapy.

## 1. Introduction

Mood disorders, such as bipolar disorder (BD), affect up to 3% of the world’s population [1]. They negatively impact the professional and social aspects of patients’ lives, leading to a significant decrease in quality of life and an increase in healthcare costs [2,3]. In addition, patients with BD have an increased risk of suicide (10–15%) [4]. Therefore, early diagnosis of BD and specific treatment will help prevent potential suicidal behavior in patients and improve their quality of life [5].

However, diagnosing BD remains challenging. The literature provides strong evidence in favor of the concept of a broad spectrum of bipolar disorders [6]. Many of the symptoms of bipolar spectrum disorders may also result from other conditions, including anxiety disorders, psychotic disorders, substance use disorders, personality disorders, etc. [7,8,9]. In addition, cardiovascular disease, diabetes, and obesity are also strongly associated with BD [10,11]. This association leads to excess cardiovascular mortality and suicide, with a loss of approximately 10–20 potential life years [9]. Delayed diagnosis and, as a result, incorrect prescription of therapy leads to worsening of the course of the disease, cognitive impairment, and reduced social functioning [12,13]. J.D. Lish et al. state that 73% of patients with BD are initially misdiagnosed, and it takes 8 years on average for patients to receive a correct diagnosis. The authors also note that in about 60% of all patients, the first episode manifests in childhood or adolescence, yet more than 50% of patients do not receive specific therapy for the next 5 years or more [14]. Approximately 75% of cases of BD manifest in late adolescence or early adulthood [15], and the disease is the fourth leading cause of the global burden of disease in adolescents and young adults [16]. 

Differential diagnosis of unipolar depression with BD is particularly challenging, and the condition is commonly underdiagnosed as a result. A total of 5–20% of patients were initially diagnosed with recurrent depression and subsequently received a revised diagnosis of BD [17]. Studies have revealed that 17% of patients observed by general practitioners and receiving prescribed long-term maintenance antidepressant therapy actually suffer from bipolar disorder. In a study of 600 patients with BD, it was found that 69% of patients initially received a different diagnosis, which changed on average about three times (the first diagnosis in 60% of patients was recurrent depression, and the second was anxiety disorder), and it took about 10 years for them to be diagnosed correctly [18].

Difficulties in diagnosing BD may be attributed to the fact that, at present, the diagnosis is made through a comprehensive clinical assessment solely based on clinical symptoms and anamnestic and follow-up data, which remain descriptive and generally do not have sufficient predictive validity [19,20]. This is due to a lack of understanding of the pathogenetic mechanisms of BD and, as a result, the absence of paraclinical markers.

Recently, highly effective methods for studying mental disorders have been developed, in particular, proteomic methods [21,22]. Proteomic analysis allows the detection of protein biomarkers associated with functional disorders involved in the pathophysiology of diseases without the need to put forward a hypothesis and limit its search area [23]. In the future, this approach could be used to help identify biomarkers of affective disorders and risks of predisposition to developing the disease later in life [24,25].

There are few published works that explore the proteomics of schizophrenia and BD, but most studies have been conducted on schizophrenia and post-mortem brain tissues [26,27,28,29,30,31]. For clinical practice, studies of biological fluids are more popular, according to which various physiological states of organs and systems, including the brain, are reflected in their composition [32,33]. Therefore, it is important to study potential biomarkers of mental disorders in an easily accessible material—blood serum [34,35,36,37,38,39,40,41]. However, all discovered proteins do not solve the problem of differential diagnosis of mental disorders due to their non-specificity. Thus, it is possible that research should focus not on the search for a specific protein but on identifying differences in the content of a set of proteins (panels) that reflect the main pathogenetic mechanisms.

Until recently, studies on the search for biomarkers of mental disorders have mainly used qualitative proteomics methods that reveal only qualitative differences in the composition of proteomes, that is, the presence or absence of specific proteins in the proteomes of the studied samples. For this reason, in recent years, quantitative proteomics has become the preferred approach for detecting proteome differences in disease [42]. However, quantitative proteomics methods using radionuclide labels are expensive, making them inconvenient for screening studies. At this point, label-free approaches may be preferable because they are simpler, more replicable, and less expensive than label-based approaches [43]. In addition, label-free semi-quantification does not limit the number of samples and conditions compared, making it suitable for longitudinal and clinical proteomics [44]. 

In this article, we searched for potential BD-specific biomarkers in serum by label-free semi-quantitative proteomics using the PeptideShaker version 2.2.9 software package. This study may be useful to future research on pathogenetic mechanisms, the development of new laboratory diagnostic criteria, and drug targets for BD and other affective disorders.

## 2. Results

In this work, mass spectrometric data of the blood serum proteome of patients with bipolar disorder (BD) and healthy individuals were analyzed in a comparative aspect. Using the SearchGUI version 3.3.12 software package and the OMSSA and X!Tandem search algorithms, protein lists were obtained for each examined patient and healthy volunteer. Tables of reliably identified proteins were formed from these lists using the PeptideShaker program. Next, the intensity values of the NSAF of reliably identified proteins in all the samples were analyzed. After comparing the proteomes of the examined individuals using a two-tailed unpaired Student’s *t*-test (FDR 0.05 and S0 = 2), proteins whose peak intensities were statistically significantly different in patients with BD and healthy individuals were identified. A list of these proteins is presented in Table 1, indicating the statistical differences according to Student’s *t*-tests, along with their NSAF values for the BD group and control patients.

The identified proteins are involved in many biological processes, including those that play an important role in the development and functioning of the central nervous system. Most of these proteins are involved in the regulation of cell adhesion, organization of the extracellular matrix, growth and migration of endothelial cells, the regulation of angiogenesis in response to a stimulus of vascular endothelial growth factor, and endothelial cell apoptosis. Proteins are also involved in the regulation of the radial mobility of neurons in the cerebral cortex controlled by glia and the growth of neuronal processes, in the regulation of cytoskeleton assembly, binding of actin filaments, and in the transmission of cell signals in glutamatergic synapses. These proteins are also involved in the processes of ubiquitination and protein catabolism. They regulate protein phosphorylation and dephosphorylation by mediating the activity of G-protein-coupled receptors; regulate the activity of GTPase, deoxycytidine triphosphatase, MAP kinase and GDP phosphatase, the ERK1 and ERK2 cascade, and other protein kinase phosphorylation enzymes; and regulate the activity of nucleoside diphosphate phosphatase and ribonucleoside triphosphate phosphatase. They participate in the regulation of cell morphogenesis, transcription, proliferation and cell differentiation, and regulation of the mitotic cycle and regulate the morphogenesis of neurons, the formation of layers in the cerebral cortex, the development of dendrites, the formation and maturation of synapses, axonogenesis, and synaptic plasticity. The identified proteins are involved in intracellular transport and the regulation of protein and vesicle transport, autophagosome assembly, transmembrane transport, the intracellular position of the Golgi complex, its assembly, and the maintenance of its structural integrity. They are also involved in the directed movement of vesicles inside the Golgi apparatus and intracellular protein transport and transport of organelles through microtubules. In addition to these functions, proteins are involved in the regulation of the immune response and differentiation of T-helpers, the regulation of cytokine production, and the apoptotic process.

**Figure 1:**
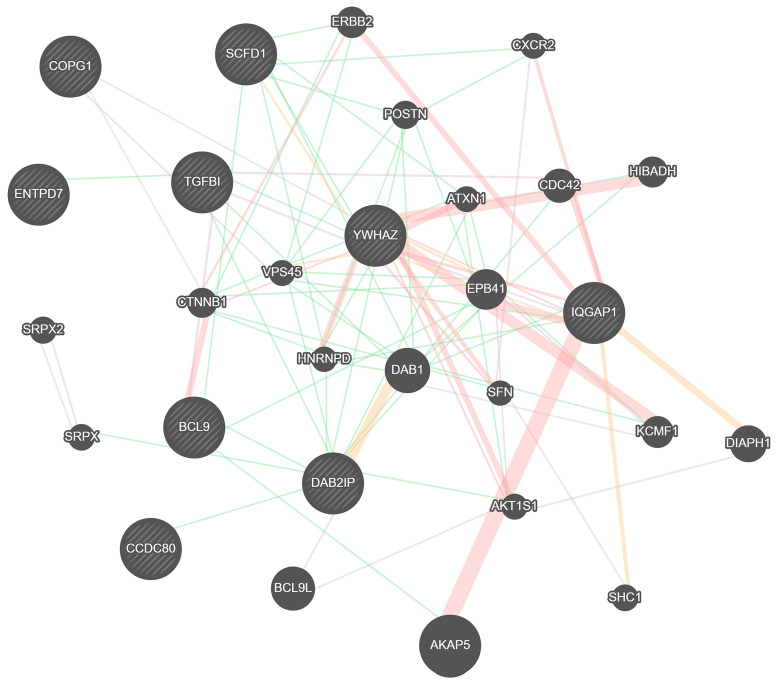
Functional network of protein gene connections that showed statistically significant differences between groups of patients with bipolar disorder and healthy individuals. Line colors indicate the following: lilac—co-expression, pink—physical interaction, orange—predicted, green—genetic interaction. The thickness of the line reflects the strength of the interaction. The tool GeneMANIA (genemania.org) was used.

Figure 1 shows a functional diagram of the relationship between the genes of the identified proteins obtained using the GeneMANIA tool [45].

The figure shows the functional gene communication network of significantly expressed proteins. The network is built based on data from studies of gene and protein expression profiles, as well as their molecular interaction pathways, from various sources such as GEO, BioGRID, Ensembl, NCBI, MGI, Pathway Commons, and others. For a complete list of GeneMANIA sources, see http://genemania.org/, accessed on 13 July 2023. The association of genes in a data set is represented by the Pearson correlation coefficient, which reflects the strength of the interaction or the reliability of the observation that they interact, and it is color-coded for the different measures.

Co-expression reflects gene expression data. Two genes are functionally related if their expression levels change equally when the conditions of the gene expression study are changed. The expression of such genes is probably regulated by the same mechanisms or by similar or interrelated pathways. For our proteins, the level of co-expression is 54.4%. Physical interaction reflects data on the protein–protein interactions of the products of related genes. For the proteins presented in the figure, the physical interaction is 26.1%. Predicted interaction, a similar indicator to physical interaction, reflects functional connections between genes, mainly protein–protein interactions, based on similar functional connections in another organism. For the proteins we identified, this indicator is 12.67%. Genetic interaction indicates a functional relationship between two genes if the level of expression of one gene entails a change in the expression of another gene. For the proteins shown in the figure, genetic interaction is quite low and amounts to 2.5%.

## 3. Discussion

In our work, we used the PeptideShaker software package, which uses several search engines on one data set at once using the target–decoy search strategy [45] for probabilistic evaluation of data processing errors. The use of this strategy to unify the peptide and spectrum matching lists (PSM) of various search engines makes it possible to increase the reliability and sensitivity of peptide detection compared with data processing methods using just one search engine [46,47]. PeptideShaker also delivers false discovery rates (FDRs) of peptides and proteins at the PSM level and allows relative quantification of peptides in different samples via label-free quantitation (LFQ) based on peak intensity or area under the curve of the detected peptide [48,49].

After using the PeptideShaker software and the statistical processing of the results, 10 differentially expressed proteins that showed significant differences between the groups of patients with bipolar disorder and healthy individuals were identified. Previously, most of these proteins were not associated with the pathogenesis of bipolar disorder. Therefore, below, we provide a description of the already known physiological and pathophysiological functions of these proteins so that, on the basis of this, we can make an assumption about their possible participation in the mechanisms of the pathogenesis of affective disorders.

B-cell CLL/lymphoma 9 protein (*BCL9*) was the most represented by the NSAF value in the BD patients in our study. This protein is involved in signal transmission through the Wnt pathway, one of the intracellular signaling pathways that regulates embryogenesis and cell differentiation [50]. Wnt signals via β-catenin and lymphoid enhancer factor 1/T-cell-specific transcription factors (LEF1/TCFs). There is ample evidence linking Wnt/β-catenin signaling to mood disorders, but the mechanism of this association is still unknown. The BCL9 gene encoding the protein we found was associated with major depressive disorder. Additionally, the classical mood stabilizer lithium can activate β-catenin by inhibiting GSK3α/β 6, which is a key enzyme in the canonical Wnt 7 pathway [51]. The Wnt pathway is a major regulator of adult hippocampal neurogenesis. Upregulation of Wnt3 enhances neurogenesis in the hippocampus in vitro and in vivo, and blockade of Wnt signaling reduces neurogenesis [52]. The genes *WNT2B* and *TCF7L2* have also been linked with BD [53,54]. In addition, astrocyte-produced Wnt is required to maintain the structural and functional integrity of BBB endothelial cells [55,56]. Thus, the protein we identified can presumably also play a role in the regulation of BBB in mental pathology. This protein can apparently also be involved in the pathogenesis of bipolar disorder, perhaps by analogy with the same mechanisms that are involved in the pathogenesis of major depressive disorder. This fact will not allow its use for the differential diagnosis of affective disorders but will undoubtedly contribute to the discovery of the mechanisms of the pathogenesis of bipolar disorder.

The second-largest NSAF in bipolar patients in our study was 14-3-3 protein zeta/delta (YWHAZ). The 14-3-3 proteins are a family of highly conserved, multifunctional proteins that are primarily expressed in the brain and are closely associated with various brain disorders, although their exact neurophysiological function is not yet fully understood [57,58]. The main function of 14-3-3 proteins is their ability to close phosphorylated regions of target proteins, thus blocking the action of phosphatases and preventing their dephosphorylation [59]. Proteins of the 14-3-3 family can also change the conformation and subcellular localization of their target proteins [60,61,62,63].

In neurons, proteins of the 14-3-3 family are present in the cytoplasmic reticulum, intracellular organelles, and the plasma membrane, where they play a functional role in cellular processes such as the differentiation, migration, and survival of neurons; neurite outgrowth; and regulation of ion channels [64,65,66]. Some of the 14-3-3 isoforms are especially abundant in synapses, where they regulate signaling and neuroplasticity [67,68,69]. The isoform found in our study, 14-3-3 ζ (14-3-3 protein zeta/delta), which is maximally represented in patients with bipolar disorder, is an activator of tyrosine and tryptophan hydroxylases, enzymes that limit the rate of synthesis of dopamine and other neurotransmitters [70,71,72]. The isoforms 14-3-3 ε and ζ are important for neuronal development [73]. The underlying mechanism involves the Ndel1/LIS1/14-3-3 protein complex, which is critical to proper neuronal migration and axon growth. Disturbances in the axonal transport of this protein complex lead to abnormal migration and development of neurons, which is associated with diseases of the nervous system, such as schizophrenia [74,75]. It was also found that gene knockout 14-3-3 ζ in mice leads to morphological changes in the brain; these mice have enlarged lateral ventricles, aberrant connectivity of mossy fibers, and reduced density of synapses and dendritic spines of the hippocampus [76,77]. These studies provide evidence of involvement 14-3-3 ζ in the formation of dendritic spines. Other genetic and post-mortem mRNA studies have identified genes for the 14-3-3 isoforms—including the gene *YWHAZ*, the encoding of which was discovered by us in the 14-3-3 ζ protein—as a potential susceptibility gene for schizophrenia [78,79,80,81]. A significant decrease in mRNA expression was also identified for 14-3-3 (isoforms β, η, ε, σ, θ, ζ) in the prefrontal cortex and for 14-3-3η in the cerebellum of patients with schizophrenia [82,83].

In addition, chromosomal regions have been identified that share common candidate genes for the risk of BD and schizophrenia. One such associated area is 22q12-13, which contains the gene 14-3-3η (*YWHAH*) [84,85,86]. For example, one meta-analysis found that *YWHAH* has a statistically significant relationship with BD [87]. In addition to 14-3-3η, other 14-3-3 isoforms are involved in BD, so reduced levels of mRNA expression have been reported in 14-3-3ε, -σ, and -ζ in brain samples of patients with BD [88].

Thus, all of the above works were performed on postmortem genetic material, theoretically proving the role of 14-3-3ζ protein in the pathogenesis of mental disorders. The potential detection of an increase in the amount of 14-3-3 protein zeta/delta in the blood serum of patients with bipolar disorder makes it a promising biomarker for BD.

For transforming growth factor-beta-induced protein ig-h3 (TGFBI), the NSAF was three times higher in patients with BD compared to that in healthy individuals. It is an extracellular matrix (ECM) protein that is involved in many physiological processes, including morphogenesis, cell adhesion and migration, angiogenesis, and inflammation [89]. The TGFBI protein connects various molecules of the extracellular matrix with each other and promotes the interaction of cells with collagen, fibronectin [90], various integrins [91], and proteoglycans such as biglycan and decorin [92]. TGFBI also triggers phosphorylation and activates several intracellular pathways, including protein kinase AKT1 (a key enzyme in the PI3K/AKT signaling pathway), focal adhesion kinase, and paxillin [93]. TGFBI is distributed in the extracellular matrices of a wide range of tissues and plays a role in the adhesion and migration of a wide range of cells, including endothelial cells [94]. Additionally, TGFBI expression is induced in reactive astrocytes of the rat cerebral cortex at sites of injury [95]. It appears that the detection of this protein indicates the activation of protective mechanisms in response to damage to the vascular endothelium, which may, in turn, indicate the modulation of the permeability of the BBB in BD. Until now, this protein has not been associated with psychiatric disorders. However, the activation of neuroinflammation and impaired BBB permeability in affective mental disorders has recently attracted more attention from researchers.

A significant difference in the NSAF value in patients with bipolar disorder and healthy individuals was also shown for ectonucleoside triphosphate diphosphohydrolase 7 (*ENTPD7*). This protein is an enzyme that catalyzes the hydrolysis of nucleoside triphosphates and diphosphates in the presence of calcium or magnesium and predominantly hydrolyzes nucleoside-5′-triphosphates. It also hydrolyzes ATP and nucleoside diphosphates to a lesser extent [96]. The functional role of this protein in the human body has not been studied practically, but it can be argued with a high degree of probability that this protein hydrolyzes nucleotides in the body and participates in important regulatory processes in purine metabolism, which plays a key role in oxidative stress, DNA damage, and cellular aging [97,98].

Until now, this protein has not been associated with mental disorders, but according to the literature, oxidative stress plays a significant role in the pathogenesis of bipolar disorder. Therefore, based on the results presented, it is very likely that the protein produced by the ENTPD7 gene also contributes to the development of BD.

The remaining six proteins, which showed a statistically significant difference in the NSAF value in patients with bipolar disorder and healthy individuals, are more frequently represented in healthy individuals and in patients with minimal peak values. These are the following proteins.

Coiled-coil domain-containing protein 80 (*CCDC80*) and TGFBI can be involved in cell adhesion and extracellular matrix assembly; however, its mechanism of action and the role of this protein in cell adhesion have not yet been studied [99]. However, its decrease in patients may also lead to an increase in BBB permeability.

Disabled homolog 2-interacting protein (*DAB2IP*) is a member of the Ras-GTPase-activating protein family and is a scaffold protein involved in the regulation of a wide range of both general and specialized signaling pathways. It is involved in processes such as innate immune response, inflammation and cell growth inhibition, apoptosis, cell survival, angiogenesis, cell migration, and maturation, and also plays a role in cell cycle regulation [100].

This protein is normally highly expressed in the vascular endothelium and functions as an inhibitor of angiogenesis and endothelial cell migration by blocking VEGFR-2 activity, so DAB2IP deficiency leads to increased VEGFR-2 mediated angiogenesis [101]. However, DAB2IP gene knockout reduces corneal and retinal angiogenesis, which is associated with positive regulation of DAB2IP endocytosis and VEGFR-3 expression [102]. Under stress of the endoplasmic reticulum caused by the accumulation of unfolded (misfolded) proteins, DAB2IP, with the participation of TNF, mediates the activation of a signaling pathway involving the enzyme apoptosis signal-regulating kinase 1 (ASK1) [103]. Activation of this mechanism under endoplasmic reticulum stress has also been described for endothelial cells [104]. TNF, together with RIP1, induces phosphorylation of DAB2IP, which in turn causes dissociation of ASK1 with its inhibitory proteins 14-3-3; this activates the signal transduction cascade for endothelial cell apoptosis [105,106,107].

DAB2IP is also highly expressed in the brain and interacts with the disabled-1 (DAB1) protein, a key mediator of the reelin pathway that controls the migration and position of neurons during development. DAB1 plays a role in neuronal migration and the growth of neurites and their processes in the developing neocortex [108] and regulates dendritic development and synapse formation in the developing cerebellum [109]. In addition, regulation of the DAB2IP gene via methylation is key to the differentiation and maturation of the central nervous system [110]. A decrease in the expression of this protein may be the key to the development of psychiatric pathology.

One other protein, adhesion G-protein-coupled receptor B1 (*ADGRB1*), is part of the G-protein-coupled family of membrane receptor protein (GPCR), which allows the nervous system to accurately respond to neurotransmitters, nucleotides, amines, peptides, cytokines and hormones, and transmit specific signals across the cell membrane [111,112]. The inherent ligand selectivity of neuronal GPCRs ensures proper integration between signal transduction pathways. Thus, about 35% of the drugs included in the FDA list act through the GPCR [113,114]. The role of GPCR in psychiatric illnesses, including schizophrenia, BD, and depression, has been discussed [115].

The ADGRB1 we discovered has a large extracellular domain (ECD) that is responsible for adhesive function [116]. This class of proteins is actively involved in the early development of the nervous system and brain [117]. The protein in the GPCR receptor allows nerve cells to transmit signals between themselves and their microenvironment and migrate to destinations to perform specific functions. Thus, it has been demonstrated that in mouse Purkinje neurons, GPCR adhesion is necessary for the formation of complex dendritic structures for synaptic connections [118]. AGRB1 regulates synaptogenesis by controlling the recruitment of the Par3/Tiam1 polarity complex to synaptic sites [119]. In the adult, ADGRB1 regulates synaptic plasticity in learning and memory in the hippocampus [120]. ADGRB1 is also involved in the formation of postsynaptic receptors that control the development of excitatory synapses [121,122]. Despite the fact that the connection of this neurospecific protein with mental disorders has already been established, its role in the pathogenesis of BD has yet to be elucidated. However, the study of its pathophysiological mechanisms is deemed to be extremely promising for the development of new drugs and methods of treatment and diagnosis of BD.

The next protein, Sec1 family domain-containing protein 1 (*SCFD1*), is a member of the Senc1/Munk 18 protein family (SM). These are vesicle-traversing proteins that interact with the SNARE integral membrane proteins that mediate the fusion of vesicles with membrane-bound compartments [123].

SCFD1 is mainly involved in transport from the endoplasmic reticulum to the Golgi apparatus by promoting membrane fusion and allowing vesicles to pass from one compartment to another [124,125]. Moreover, it plays a significant protective role in response to oxidative stress, participating in the mechanisms of protection of the endoplasmic reticulum from morphological changes associated with oxidative stress and preventing cell death. SCFD1 has been proven to be closely associated with the pathogenesis of Parkinson’s disease and amyotrophic lateral sclerosis (ALS) [126]. This is how the association of the rs10139154 polymorphism of the SCFD1 gene with the risk of developing ALS is described [127]. The mechanisms underlying possible neurodegeneration have not yet been fully elucidated, although it has been demonstrated that ALS patients have impaired endosomal transport function and dysfunction of the Golgi network [128]. These effects are exacerbated under conditions of stress, leading to neurodegeneration [129,130]. In addition, in Alzheimer’s disease and ALS, impairment of endocytic migration was revealed [131,132]. In these neurodegenerative diseases, pathogenetic mechanisms involving SCFD1 have also been identified, leading to a disruption in the mechanism of axonal transport of proteins to the synaptic ending, which is regulated by neurofilaments [133,134,135]. Thus, until now, this protein has been associated with neurodegenerative diseases, and its connection with mental disorders has been identified for the first time in this study. Based on the mechanisms described above, a decrease in its amount in patients with bipolar disorder causes a decrease in protection against OS, which in turn triggers a vicious circle (pathway): oxidative stress–inflammation–oxidative stress. This presumably leads to both the development of chronic affective disorders and the development of neurodegenerative diseases.

Ras GTPase-activating-like protein IQGAP1 (*IQGAP1*) is a member of the Ras superfamily of small GTPases [136], a large family of monomeric G proteins with structural and functional homology to the Gα subunits of heterotrimeric G proteins. These proteins play a crucial role in regulating the dynamics and assembly of the actin cytoskeleton in the leading processes of migrating neurons, directing and coordinating changes in both actin filaments and microtubules. IQGAP1 binds to and alters the function of several proteins, including actin, E-cadherin, beta-catenin, Cdc42, phospholipase C epsilon 1 (PLCe1), Lissencephaly-1 homolog (Lis1), and Ras-related C3 botulinum toxin substrate 1 (Rac1). 

Lis1 regulates the activity of motor protein dynein by mediating microtubule glide during nuclear migration during cell division [137,138]. Lis1 is known to be involved in the localization of GABAergic synaptic vesicles in the ventral nerve trunk [139] and the differentiation of neurons [140]. Defects in the Lis1 gene disrupt neuronal migration, causing severe lissencephaly, a complex disorder of brain function caused by anomalies in neurogenesis. Although the role of Lis1 in this process is not fully understood, Lis1 deficiency is known to cause dysregulation of the Ras GTPases Cdc42, Rac1, and RhoA family proteins and subsequent cytoskeletal actin defects. Here, we note that IQGAP1 may be actively involved in the reorganization of the actin cytoskeleton. IQGAP1 is a substrate for the PLCe1 protein kinase, which catalyzes the phosphorylation of Ser-1443 to promote neurite outgrowth [141,142]. Thus, IQGAP1 may play a role in cell cycle regulation by promoting neurite outgrowth, neurogenesis, neuronal migration, and dendritic outgrowth. A decrease in the expression of this protein may be associated with the development of bipolar disorder.

Coatomer subunit gamma-1 (*COPG1*) is a cytosolic protein that, as part of the coatomer complex (COPI), binds to dilysin proteins and Golgi vesicles that are not covered with clathrin. COPI is required for the budding of vesicles from the Golgi membranes and the transport of dilysin-labeled proteins from the endoplasmic reticulum and also affects the structural integrity of the Golgi apparatus and recycling of LDL receptors on the plasma membrane [143,144]. 

The role of the COPG1 protein in the development of mental illness has not yet been studied. It has been reported that the COPI complex may play a role in Alzheimer’s disease, as it is involved in the localization of the amyloid precursor protein, and downregulation of the COPI delta subunit (δ-COP) expression leads to a decrease in amyloid plaques and improved memory in mice [145]. It has also been shown in a cell model on mouse neuronal progenitors that Copg1, an analog of human COPG1, is required for the proper differentiation and maturation of neurons. Impairment of this gene leads to morphological changes in the Golgi apparatus and disruption of the formation of neuronal processes [146]. Reduced expression of this protein significantly reduces neuroplasticity and, under certain conditions, likely leads to the development of bipolar disorder.

Eight proteins presented in our study were shown to be associated with BD for the first time and were not previously associated with the pathogenesis of BD. Two of the previously identified proteins with significant differences had already been shown to be associated with BD. All ten differentially expressed proteins were found to be neurospecific to one degree or another. Figure 1 shows a functional diagram of the relationships between the genes of the identified proteins, showing a large number of different connections. The proteins we identified showed a high level of co-expression of 54.4%, which indicates a close functional connection between the proteins and, most likely, mutual influence in the pathway of bipolar disorder.

Thus, the use of the PeptideShaker software package in this work made it possible to conduct a semi-quantitative analysis of the protein spectrum without using a labeled standard and to approximately estimate the protein levels of comparison groups without using expensive quantitative proteomics methods. From this work, it was concluded that PeptideShaker is an easy-to-use tool for analyzing mass spectrometry data at the initial exploratory stage of a study. The tool is suitable for both the analysis and interpretation of primary data and for the re-analysis and comparison of data from different experiments obtained over different periods of time. The most important reason we chose this tool is the ability to work with the combined results of several identification algorithms on a single set of input data. In this case, the program uses spectrum files in the general Mascot format (MGF) (which can be obtained from any MS spectrum file) and allows one to load custom databases of protein sequences in the FASTA file format and analyze experiments performed over a long time under the same conditions using one protein database. Also, to increase the reliability of search results, PeptideShaker uses a “target-bait” approach, in which the protein database is supplemented with false sequences; in our case, the reverse sequences of all the proteins were used, which reduced the rate of false discoveries.

## 4. Materials and Methods

### 4.1. Patient Characteristics

In the present work, patients with bipolar disorder treated in the Affective States Department of Mental Health Research Institute of Tomsk National Research Medical Center were examined. This study used a cross-sectional design. The group of patients with BD included 8 individuals. The patients’ age (Me [Q25; Q75]) was 44 [38; 48] years, and the disease duration was 8 [4; 12] years. The age of disease manifestation was 33 [22; 45] years; the average number of experienced episodes was 4 [2; 5]. Most of the patients were treated for a current depressive episode of varying severity with diagnoses according to ICD-10: F31.3—4 individuals, F31.5—2 individuals. Additionally, this group included patients with a current mixed affective episode (F31.6—2 individuals).

The criteria for inclusion in the group of examined persons were the presence of an established diagnosis of bipolar disorder (F31) based on the ICD-10 criteria, age from 18 to 55 years, the absence of acute somatic pathology, and the presence of a signed informed consent form for participation in this study.

The control group consisted of 7 mentally healthy individuals aged 42 [37; 45] years without signs of acute somatic diseases at the time of the examination. For the control group, the inclusion criteria for this study were age from 18 to 55 years, the absence of somatic and mental diseases, and the presence of written informed consent to participate in this study.

Exclusion criteria were age over 55 years, the presence of somatic pathology in the acute stage, epilepsy, alcoholism, drug addiction, or any other mental disorder not included in this study.

This study was conducted according to the guidelines of the Declaration of Helsinki and approved by the Ethical Committee of Mental Health Research Institute at Tomsk National Research Medical Center of the Russian Academy of Sciences No. 163 from 12 May 2023 (No. 163/3.2023).

### 4.2. Sample Preparation

Venous blood was taken in the morning on an empty stomach in sample tubes (Becton Dickinson Vacutainer, BD, Vianen, the Netherlands) containing a clot activator. Serum was separated from blood via centrifugation for 20 min at 2000× *g* in a Digicen 21R centrifuge (Orto Alresa, Madrid, Spain). The serum was then divided into aliquots and stored at −80 °C.

### 4.3. Affinity Chromatography

Serum samples were diluted 5-fold with sodium phosphate buffer (phosphate-buffered saline—PBS), centrifuged at 16,000 rpm for 1 min in a Zentrifuge Z 36 HK centrifuge (Hermle Labortechnik Gmbh, Wehingen, Germany) at 4 °C, and filtered through a standard filter Filtropur S (Sarstedt, Nümbrecht, Germany) with a diameter of 22 microns. The resulting supernatant was passed through a Multiple Affinity Removal Column Human 14 (4.6 × 100 mm), Agilent, Santa Clara, CA, USA) for affinity binding and removal of 14 major proteins: albumin, IgG, IgA, transferrin, haptoglobin, antitrypsin, fibrinogen, alpha2-macroglobulin, alpha1-acid glycoprotein, IgM, apolipoprotein AI, apolipoprotein AII, complement C3, and transthyretin using an Agilent 1200 series HPLC system.

The resulting samples were concentrated via ultrafiltration through 3 kDa Microcon^®^ centrifuge ultrafilters (Millipore, Molsheim, France) at 14,000 rcf for 15 min at 20 °C. The protein concentration was measured based on the absorbance at 280/260 nm using a Varioskan LUX spectrophotometer (Thermo Scientific, Waltham, MA, USA) located at the core facility Medical Genomics at Tomsk National Research Center.

### 4.4. One-Dimensional Laemmli PAG Electrophoresis

The staining agent (2 mL of glycerol, 2 mL of 0.5 M Tris-HCl pH 6.8, 750 μL of deionized water, 10 μL of 4% SDS, 30 μL of β-mercaptoethanol, bromophenol blue) was added to the samples after purification, as described above, in a ratio of 1: 2. The samples were heated at 95 °C for 5 min and briefly centrifuged to precipitate the condensate. Samples containing 30 μg of total protein were added to the gel and subjected to electrophoresis in a polyacrylamide gel with sodium dodecyl sulfate according to the Laemmli method in 12% PAGE [147]. To control the migration of proteins in the gel and further calculate the molecular weights, a set of 14 recombinant highly purified unstained PageRuler proteins 10–200 kDa (Fermentas, Waltham, MA, USA) was used. Electrophoresis was performed in a Protean II xi Cell (Bio-Rad, Hercules, CA, USA) at a voltage of 150–180 V, powered by a PowerPac™ Universal Power Supply (Bio-Rad). The gels were stained with Coomassie brilliant blue G250 (0.1% Coomassie brilliant blue G250, 40% C_2_H_5_OH, 10% CH_3_COOH), and washing was carried out in a solution of 5% CH_3_COOH and 50% C_2_H_5_OH until the stained bands were visualized.

The molecular weights corresponding to the stained protein bands in the gel were automatically calculated using the iBright Imaging Systems FL1500 gel documentation system (Thermo Fisher Scientific) at the base of the Core Facility “Medical Genomics” Tomsk NRMC with the accompanying software relative to molecular weights of protein standards (Broad range, Fermentas, Thermo Fisher Scientific). Statistically significant differences in the distribution of proteins between patients and healthy individuals were determined using Fisher’s exact test with Yates’ correction. Protein bands that differed statistically significantly between the groups of patients with BD and healthy individuals were subjected to further analysis. These bands were manually cut out of the gel (size ~3 mm) using a scarifier and placed in microtubes. From 8 to 24 samples from different bands from each patient were included in this study.

Next, the Coomassie G250 dye was removed by washing three times with 50 mM NH_4_HCO_3_ in 50% acetonitrile for 10–15 min on a shaker until complete discoloration was achieved. Then, the samples were lyophilized for 45 min at 40 °C.

### 4.5. Trypsinolysis

Trypsinolysis was performed using trypsin modified for sequencing (#V511A, Promega, Madison, WI, USA) diluted with the supplied solution (50 mM CH_3_COOH) and then sequentially with 50 mM NH_4_HCO_3_, pH = 8, to a concentration of 0.01–0.025 mcg/mL. After that, 20 µL of trypsin solution was added to each sample and incubated at 4 °C for 1 h to swell the gel. Then, the samples were incubated at 37 °C for 18 h to achieve trypsinolysis. After the reaction was complete, 25 mM NH_4_HCO_3_ was added to each sample and vortexed, and the supernatants were placed in separate tubes. Next, the peptide mixtures were extracted from the gels with 50% acetonitrile in 5% formic acid; the procedure was repeated three times. The extracts were lyophilized and frozen.

### 4.6. Mass Spectrometry Analysis

Mass spectrometric analysis was performed based on the Advanced Mass Spectrometry Core Facility at the Skolkovo Institute of Science and Technology (Moscow, Russia) according to the Rusanov protocol with minimal changes [148].

The resulting peptide samples were analyzed using an Ultimate 3000 Nano LC HPLC system (Thermo Scientific, Rockwell, IL, USA) coupled to a Q Exactive HF-X hybrid quadrupole—Orbitrap (Thermo Fisher Scientific).

Peptide separation was performed on a C18 column with an inner diameter of 75 μm and a length of 150 mm (Acclaim^®^ PepMap™ RSLC, Thermo Fisher Scientific, Rockwell, IL, USA). One microliter of sample, equivalent to one microgram of peptides, was loaded directly onto the column and equilibrated isocratically with mobile phase C (2% acetonitrile, 0.1% formic acid). The peptides were then eluted using a linear gradient of 5 to 55% solution B (0.1% formic acid and 80% acetonitrile) at a flow rate of 0.3 μL/min for 75 min, then for another 6 min to reach 99% solution B. Before the next sample was loaded, a 10 min wash with 99% solution B and a 7 min re-equilibration of the column with solution A (0.1% formic acid) was performed.

Then, the eluting peptides were loaded into the mass spectrometer through a capillary at a temperature of 240 °C and an emitter voltage of 2.1 kV.

Samples were analyzed in triplicate in full MS mode followed by a single DDA MS2. Mass spectra were obtained in the mass range 320–1500 *m*/*z* with a resolution of 120,000 (MS). Precursor ions were fragmented in the HCD mode (Higher-Energy Collision Dissociation). Tandem mass spectra of the fragments were obtained at a resolution of 15,000 (MS/MS) in the range from 140 *m*/*z* to the *m*/*z* value determined by the charge state of the precursor, but not more than 2000 *m*/*z*. The maximum accumulation time of precursor ions was 50 ms, and that of fragment ions was 100 ms. The target fill value of the automatic gain control (AGC) for precursor and fragment ions was set to 1 × 10^6^ and 2 × 10^5^, respectively.

An isolation intensity threshold of 50,000 arbitrary units was defined for precursor selection, and up to 20 of the best precursors were selected for fragmentation at a normalized collision energy (NCE) of 30. The precursor ion isolation width was 2 *m*/*z*. Precursors with a charge state of 1+ and more than 5+ were rejected, and all measured precursors were dynamically excluded from the launch of the subsequent MS/MS for 20 s.

### 4.7. Protein Identification and Statistical Analysis

Peak lists obtained from MS/MS spectra were identified using OMSSA version 2.1.9 [149] and X!Tandem version X! Tandem Vengeance [150]. The search was conducted using SearchGUI version 3.3.12 [151].

Protein identification was conducted against a concatenated target/decoy version of the UniProtKB [152] (release 2023_03; 20,423 (target) sequences) database considering the species *Homo sapiens*). The decoy sequences were created by reversing the target sequences in SearchGUI. The identification settings were as follows: trypsin, with a maximum of 1 missed cleavage 10.0 ppm as MS1 and 0.05 Da as MS2 tolerances; variable modifications: deamidation of N (+0.984016 Da), deamidation of Q (+0.984016 Da), oxidation of M (+15.994915 Da), and propionamide of C (+71.037114 Da). Variable modifications during refinement procedure: acetylation of protein N-term (+42.010565 Da), pyrrolidone from E (−18.010565 Da), and pyrolidone from Q (−17.026549 Da). The rest of the parameters were set to the default values.

Peptides and proteins were inferred from the spectrum identification results using PeptideShaker version 2.2.9 [153]. Peptide–Spectrum matches (PSMs), peptides, and proteins were validated at a 1.0% false discovery rate (FDR) estimated using the decoy hit distribution. Proteins were considered reliably identified if at least two of their peptides could be identified. Post-translational modification localizations were scored using the D-score [154] and the phosphoRS score [155] with a threshold of 95.0, as implemented in the compomics-utilities package [156]. A phosphoRS score above this threshold was considered a confident localization.

The mass spectrometry data, along with the identification results, were submitted to the Figshare repository. These data can be accessed at https://figshare.com/articles/journal_contribution/Analysis_of_Mass_Spectrometric_Data_of_Proteins_from_Serum_in_Patients_with_Bipolar_Disorder_and_and_ealthy_Individuals_Using_the_PeptideShaker_Software/24033309 (posted date 25 August 2023).

An unlabeled analysis based on the normalized spectral abundance factor (NSAF) was used to detect differences in the relative abundance of detected proteins between study groups [157].

The NSAF depends on the number of identified peptide spectra for each protein and makes it possible to compare the contents of individual proteins in several independent samples, which allows it to be widely used in semi-quantitative proteomics [158,159,160]. This index showed good specificity and sensitivity for low-protein samples, which may be useful for determining minor proteins in serum [159,161,162,163]. Statistically significant differences in the mean NSAF values for each protein in the study group were assessed using a two-tailed unpaired Student’s *t*-test with Bonferroni correction (*p* = 0.05) in the Statistica 10.0 software package (StatSoft, Hamburg, Germany).

## 5. Conclusions

Following the application of the PeptideShaker software package, during the comparison of the serum proteomes of patients with bipolar disorder and healthy individuals, several proteins were identified that are likely to be involved in the pathogenetic processes of BD. When comparing the proteomes of the blood serum of patients with bipolar disorder and healthy individuals, 10 proteins showed significant differences in NSAF values. Of these, four proteins were predominantly present in BD patients with the maximum NSAF value: 14-3-3 protein zeta/delta (NSAF = 0.009346; *p* = 0.06); ectonucleoside triphosphate diphosphohydrolase 7 (NSAF = 0.002105; *p* = 0.02); transforming growth factor-beta-induced protein ig-h3 (NSAF = 0.005941; *p* = 0.02); and B-cell CLL/lymphoma 9 protein (NSAF = 0.018721; *p* = 0.01). Meanwhile, another six proteins were minimally represented in the serum of BD patients (NSAF ≈ 0.00001). In total, 8 out of 10 proteins were found to be associated with BD for the first time. As these proteins are generally considered to be neurospecific, we believe there is great potential for further studies of the role of these proteins in the pathogenesis of BD and their quantitative contents in a larger number of patients. Conducting such studies will help with the development of new paraclinical criteria for the differential diagnosis of bipolar disorder, among other mental disorders, and the discovery of new targets for BD drug therapy. This will significantly improve the quality and timeliness of BD diagnosis, as well as the choice of the optimal therapy algorithm, significantly improving the quality of life and decreasing the risk of suicidal behavior in these patients. ***Limitations.*** This study used a cross-sectional design to recruit patients. Because this was a pilot study, the cohort size was limited. In this study, we tested the PeptideShaker tool to perform a rapid semi-quantitative comparative analysis of serum proteomes involving a large number of proteins.

## Figures and Tables

**Table 1:** Statistically significant differences found between the group of patients with bipolar disorder and the control group.

Uniprot Code	Protein Name	Gene	NSAF BD Mean	NSAF Control Mean	Student’s *t*-Test, *p*
Q15582	Transforming growth factor-beta-induced protein ig-h3	*TGFBI*	0.005941	0.001980	0.0342
Q5VWQ8	Disabled homolog 2-interacting protein	*DAB2IP*	0.000014	0.004689	0.01055
Q76M96	Coiled-coil domain-containing protein 80	*CCDC80*	0.000015	0.001359	0.01064
O00512	B-cell CLL/lymphoma 9 protein	*BCL9*	0.018721	0.000029	0.01092
Q9Y678	Coatomer subunit gamma-1	*COPG1*	0.000018	0.002829	0.01281
P46940	Ras GTPase-activating-like protein IQGAP1	*IQGAP1*	0.0000019	0.000765	0.01446
Q9NQZ7	Ectonucleoside triphosphate diphosphohydrolase 7	*ENTPD7*	0.002105	0.000012	0.01655
O14514	Adhesion G-protein-coupled receptor B1	*ADGRB1*	0.000015	0.003524	0.02106
Q8WVM8	Sec1 family domain-containing protein	*SCFD1*	0.000017	0.001866	0.00486
P63104	14-3-3 protein zeta/delta	*YWHAZ*	0.009346	0.000026	0.005644

## Data Availability

The data presented in this study are available on request from the corresponding author.

## References

[1] Ferrari A.J., Stockings E., Khoo J.P., Erskine H.E., Degenhardt L., Vos T., Whiteford H.A. (2016). The prevalence and burden of bipolar disorder: Findings from the Global Burden of Disease Study 2013. Bipolar Disord..

[2] Carvalho A.F., Firth J., Vieta E. (2020). Bipolar Disorder. N. Engl. J. Med..

[3] Hirschfeld R.M., Calabrese J.R., Weissman M.M., Reed M., Davies M.A., Frye M.A., Keck P.E., Lewis L., McElroy S.L., McNulty J.P. (2003). Screening for bipolar disorder in the community. J. Clin. Psychiatry.

[4] Cavazzoni P., Grof P., Duffy A., Grof E., Müller-Oerlinghausen B., Berghöfer A., Ahrens B., Zvolsky P., Robertson C., Davis A. (2007). Heterogeneity of the risk of suicidal behavior in bipolar-spectrum disorders. Bipolar Disord..

[5] Kamali M., Reilly-Harrington N.A., Chang W.C., McInnis M., McElroy S.L., Ketter T.A., Shelton R.C., Deckersbach T., Tohen M., Kocsis J.H. (2019). Bipolar depression and suicidal ideation: Moderators and mediators of a complex relationship. J. Affect. Disord..

[6] Angst J., Gamma A. (2002). A new bipolar spectrum concept: a brief review. Bipolar Disord..

[7] Simutkin G.G., Bokhan N.A., Ivanova S.A., Ovchinnikov A.A., Aksenov M.M. (2023). Probabilistic Diagnosis of Bipolar Adjective Disorder: Modern Approaches, Possibly and Restrictions.

[8] Vieta E., Berk M., Schulze T.G., Carvalho A.F., Suppes T., Calabrese J.R., Gao K., Miskowiak K.W., Grande I. (2018). Bipolar disorders. Nat. Rev. Dis. Primers.

[9] McIntyre R.S., Berk M., Brietzke E., Goldstein B.I., López-Jaramillo C., Kessing L.V., Malhi G.S., Nierenberg A.A., Rosenblat J.D., Majeed A. (2020). Bipolar disorders. Lancet.

[10] Teixeira A.L., Colpo G.D., Fries G.R., Bauer I.E., Selvaraj S. (2019). Biomarkers for bipolar disorder: Current status and challenges ahead. Expert. Rev. Neurother..

[11] Weiner M., Warren L., Fiedorowicz J.G. (2011). Cardiovascular morbidity and mortality in bipolar disorder. Ann. Clin. Psychiatry Off. J. Am. Acad. Clin. Psychiatr..

[12] Knežević V., Nedić A. (2013). Influence of misdiagnosis on the course of bipolar disorder. Eur. Rev. Med. Pharmacol. Sci..

[13] Schaffer A., Isometsa E.T., Tondo L., Moreno D.H., Turecki G., Reis C., Cassidy F., Sinyor M., Azorin J.M., Kessing L.V. (2015). International Society for Bipolar Disorders Task Force on Suicide: Meta-analyses and meta-regression of correlates of suicide attempts and suicide deaths in bipolar disorder. Bipolar Disord..

[14] Lish J.D., Dime-Meenan S., Whybrow P.C., Price R.A., Hirschfeld R.M. (1994). The national Depressive and Manic-Depressive Association (DMDA) survey of bi-polai members. J. Affect. Disord..

[15] Geoffroy P.A., Leboyer M., Scott J. (2015). Predicting bipolar disorder: what can we learn from prospective cohort studies?. Encephale.

[16] Gore F.M., Bloem P.J., Patton G.C., Ferguson J., Joseph V., Coffey C., Sawyer S.M., Mathers C.D. (2011). Global burden of disease in young people aged 10–24 years: A systematic analysis. Lancet.

[17] Bebbington P., Ramana R. (1995). The epidemiology of bipolar affective disorder. Soc. Psychiatry Psychiatr. Epidemiol..

[18] Hirschfeld R.M., Lewis L., Vornik L.A. (2003). Perceptions and impact of bipolar disorder: How far have we really come? Results of the National Depressive and Manic-Depressive Association 2000 survey of individuals with bipolar disorder. J. Clin. Psychiatry.

[19] Geoffroy P.A., Scott J. (2017). Prodrome or risk syndrome: What’s in a name?. Int. J. Bipolar Disord..

[20] McIntyre R.S., Cha D.S., Jerrell J.M., Swardfager W., Kim R.D., Costa L.G., Baskaran A., Soczynska J.K., Woldeyohannes H.O., Mansur R.B. (2014). Advancing biomarker research: Utilizing ‘Big Data’ approaches for the characterization and prevention of bipolar disorder. Bipolar Disord..

[21] de Jesus J.R., de Campos B.K., Galazzi R.M., Martinez J.L., Arruda M.A. (2015). Bipolar disorder: Recent advances and future trends in bioanalytical developments for biomarker discovery. Anal. Bioanal. Chem..

[22] Saia-Cereda V.M., Cassoli J.S., Martins-de-Souza D., Nascimento J.M. (2017). Psychiatric disorders biochemical pathways unraveled by human brain proteomics. Eur. Arch. Psychiatry Clin. Neurosci..

[23] Martins-de-Souza D., Maccarrone G., Wobrock T., Zerr I., Gormanns P., Reckow S., Falkai P., Schmitt A., Turck C.W. (2010). Proteome analysis of the thalamus and cerebrospinal fluid reveals glycolysis dysfunction and potential biomarkers candidates for schizophrenia. J. Psychiatr. Res..

[24] Taurines R., Dudley E., Grassl J., Warnke A., Gerlach M., Coogan A.N., Thome J. (2011). Proteomic research in psychiatry. J. Psychopharmacol..

[25] Martins-de-Souza D. (2013). Biomarkers for psychiatric disorders: where are we standing?. Dis. Markers.

[26] Smirnova L., Seregin A., Boksha I., Dmitrieva E., Simutkin G., Kornetova E., Savushkina O., Letova A., Bokhan N., Ivanova S. (2019). The difference in serum proteomes in schizophrenia and bipolar disorder. BMC Genom..

[27] Dmitrieva E., Smirnova L., Seregin A., Zgoda V., Semke A., Ivanova S. (2022). Proteomic profile of serum from patients with schizophrenia spectrum disorders. PeerJ.

[28] Silva-Costa L.C., Carlson P.T.C., Guest P.C., de Almeida V., Martins-de-Souza D. (2019). Proteomic Markers for Depression. Adv. Exp. Med. Biol..

[29] Novikova S.I., He F., Cutrufello N.J., Lidow M.S. (2006). Identification of protein biomarkers for schizophrenia and bipolar disorder in the postmortem prefrontal cortex using SELDI-TOF-MS ProteinChip profiling combined with MALDI-TOF-PSD-MS analysis. Neurobiol. Dis..

[30] Beasley C.L., Pennington K., Behan A., Wait R., Dunn M.J., Cotter D. (2006). Proteomic analysis of the anterior cingulate cortex in the major psychiatric disorders: Evidence for disease-associated changes. Proteomics.

[31] Behan A.T., Byrne C., Dunn M.J., Cagney G., Cotter D.R. (2009). Proteomic analysis of membrane microdomain-associated proteins in the dorsolateral prefrontal cortex in schizophrenia and bipolar disorder reveals alterations in LAMP, STXBP1 and BASP1 protein expression. Mol. Psychiatry.

[32] Guest P.C., Chan M.K., Gottschalk M.G., Bahn S. (2014). The use of proteomic biomarkers for improved diagnosis and stratification of schizophrenia patients. Biomark. Med..

[33] Comes A.L., Papiol S., Mueller T., Geyer P.E., Mann M., Schulze T.G. (2018). Proteomics for blood biomarker exploration of severe mental illness: Pitfalls of the past and potential for the future. Transl. Psychiat..

[34] Ding Y.H., Guo J.H., Hu Q.Y., Jiang W., Wang K.Z. (2015). Protein Biomarkers in Serum of Patients with Schizophrenia. Cell Biochem. Biophys..

[35] Chen J., Huang C., Song Y., Shi H., Wu D., Yang Y., Rao C., Liao L., Wu Y., Tang J. (2015). Comparative proteomic analysis of plasma from bipolar depression and depressive disorder: identification of proteins associated with immune regulatory. Protein Cell.

[36] Steiner J., Guest P.C. (2017). A Clinical Study Protocol to Identify Serum Biomarkers Predictive of Response to Antipsychotics in Schizophrenia Patients. Adv. Exp. Med. Biol..

[37] Seregin A.A., Smirnova L.P., Dmitrieva E.M., Vasil’eva S.N., Semke A.V., Ivanova S.A. (2020). Glutamate Level’s in Blood Serum of Patients with Schisophrenic Spectrum and Bipolar Affective Disorder. Psikhiatriya.

[38] Ivanova S.A., Smirnova L.P., Shchigoreva Y.G., Semke A.V., Bokhan N.A. (2015). Serum Glutathione in Patients with Schizophrenia in Dynamics of Antipsychotic Therapy. Bull. Exp. Biol. Med..

[39] Sabherwal S., English J.A., Föcking M., Cagney G., Cotter D.R. (2016). Blood biomarker discovery in drug-free schizophrenia: the contribution of proteomics and multiplex immunoassays. Expert. Rev. Proteom..

[40] Seregin A.A., Smirnova L.P., Dmitrieva E.M., Boksha I.S., Savushkina O.K., Simutkin G.G., Ivanova S.A. (2022). Correlations between clinical features of bipolar affective disorder and serum concentrations of ANKRD12 gene product, coagulation factor XIII, and cadherin 5. Zhurnal Nevrol. I Psikhiatrii Im. SS Korsakova.

[41] Rhee S.J., Shin D., Shin D., Song Y., Joo E.J., Jung H.Y., Roh S., Lee S.H., Kim H., Bang M. (2023). Latent class analysis of psychotic-affective disorders with data-driven plasma proteomics. Transl. Psychiatry.

[42] Bantscheff M., Lemeer S., Savitski M.M., Kuster B. (2012). Quantitative mass spectrometry in proteomics: Critical review update from 2007 to the present. Anal. Bioanal. Chem..

[43] Latosinska A., Vougas K., Makridakis M., Klein J., Mullen W., Abbas M., Stravodimos K., Katafigiotis I., Merseburger A.S., Zoidakis J. (2015). Comparative Analysis of Label-Free and 8-Plex iTRAQ Approach for Quantitative Tissue Proteomic Analysis. PLoS ONE.

[44] Martins-de-Souza D., Guest P.C., Vanattou-Saifoudine N., Harris L.W., Bahn S. (2011). Proteomic technologies for biomarker studies in psychiatry: advances and needs. Int. Rev. Neurobiol..

[45] Elias J.E., Gygi S.P. (2007). Target-decoy search strategy for increased confidence in large-scale protein identifications by mass spectrometry. Nat. Methods.

[46] Shteynberg D., Nesvizhskii A.I., Moritz R.L., Deutsch E.W. (2013). Combining results of multiple search engines in proteomics. Mol. Cell Proteom..

[47] Nesvizhskii A.I. (2010). A survey of computational methods and error rate estimation procedures for peptide and protein identification in shotgun proteomics. J. Proteom..

[48] Krey J.F., Wilmarth P.A., Shin J.B., Klimek J., Sherman N.E., Jeffery E.D., Choi D., David L.L., Barr-Gillespie P.G. (2014). Accurate label-free protein quantitation with high- and low-resolution mass spectrometers. J. Proteome Res..

[49] Välikangas T., Suomi T., Elo L.L. (2018). A comprehensive evaluation of popular proteomics software workflows for label-free proteome quantification and imputation. Brief. Bioinform..

[50] Kramps T., Peter O., Brunner E., Nellen D., Froesch B., Chatterjee S., Murone M., Züllig S., Basler K. (2002). Wnt/wingless signaling requires BCL9/legless-mediated recruitment of pygopus to the nuclear beta-catenin-TCF complex. Cell.

[51] Wiese K.E., Nusse R., van Amerongen R. (2018). Wnt signalling: conquering complexity. Development.

[52] Lie D.C., Colamarino S.A., Song H.J., Désiré L., Mira H., Consiglio A., Lein E.S., Jessberger S., Lansford H., Dearie A.R. (2005). Wnt signalling regulates adult hippocampal neurogenesis. Nature.

[53] Zandi P.P., Belmonte P.L., Willour V.L., Goes F.S., Badner J.A., Simpson S.G., Gershon E.S., McMahon F.J., DePaulo J.R., Potash J.B. (2008). Association study of Wnt signaling pathway genes in bipolar disorder. Arch. Gen. Psychiatry.

[54] Cuellar-Barboza A.B., Winham S.J., McElroy S.L., Geske J.R., Jenkins G.D., Colby C.L., Prieto M.L., Ryu E., Cunningham J.M., Frye M.A. (2016). Accumulating evidence for a role of TCF7L2 variants in bipolar disorder with elevated body mass index. Bipolar Disord..

[55] Guérit S., Fidan E., Macas J., Czupalla C.J., Figueiredo R., Vijikumar A., Yalcin B.H., Thom S., Winter P., Gerhardt H. (2021). Astrocyte-derived Wnt growth factors are required for endothelial blood-brain barrier maintenance. Prog. Neurobiol..

[56] Gastfriend B.D., Nishihara H., Canfield S.G., Foreman K.L., Engelhardt B., Palecek S.P., Shusta E.V. (2021). Wnt signaling mediates acquisition of blood–brain barrier properties in naïve endothelium derived from human pluripotent stem cells. eLife.

[57] Baxter H.C., Fraser J.R., Liu W.G., Forster J.L., Clokie S., Steinacker P., Otto M., Bahn E., Wiltfang J., Aitken A. (2002). Specific 14-3-3 isoform detection and immunolocalization in prion diseases. Biochem. Soc. Trans..

[58] Jones D.H., Ley S., Aitken A. (1995). Isoforms of 14-3-3 protein can form homo- and heterodimers in vivo and in vitro: implications for function as adapter proteins. FEBS Lett..

[59] Obsil T., Obsilova V. (2011). Structural basis of 14-3-3 protein functions. Semin. Cell Dev. Biol..

[60] Brunet A., Kanai F., Stehn J., Xu J., Sarbassova D., Frangioni J.V., Dalal S.N., DeCaprio J.A., Greenberg M.E., Yaffe M.B. (2002). 14-3-3 transits to the nucleus and participates in dynamic nucleocytoplasmic transport. J. Cell Biol..

[61] Sluchanko N.N., Gusev N.B. (2017). Moonlighting chaperone-like activity of the universal regulatory 14-3-3 proteins. FEBS J..

[62] Bridges D., Moorhead G.B. (2005). 14-3-3 proteins: a number of functions for a numbered protein. Sci. STKE.

[63] Jin J., Smith F.D., Stark C., Wells C.D., Fawcett J.P., Kulkarni S., Metalnikov P., O’Donnell P., Taylor P., Taylor L. (2004). Proteomic functional, and domain-based analysis of in vivo 14-3-3 binding proteins involved in cytoskeletal regulation and cellular organization. Curr. Biol..

[64] van Hemert M.J., Steensma H.Y., van Heusden G.P. (2001). 14-3-3 proteins: key regulators of cell division, signalling and apoptosis. Bioessays.

[65] Freeman A.K., Morrison D.K. (2011). 14-3-3 Proteins: diverse functions in cell proliferation and cancer progression. Semin. Cell Dev. Biol..

[66] Gardino A.K., Yaffe M.B. (2011). 14-3-3 proteins as signaling integration points for cell cycle control and apoptosis. Semin. Cell Dev. Biol..

[67] Baxter H.C., Liu W.G., Forster J.L., Aitken A., Fraser J.R. (2002). Immunolocalisation of 14-3-3 isoforms in normal and scrapie-infected murine brain. Neuroscience.

[68] Broadie K., Rushton E., Skoulakis E.M., Davis R.L. (1997). Leonardo, a Drosophila 14-3-3 protein involved in learning, regulates presynaptic function. Neuron.

[69] Zhou Y., Schopperle W.M., Murrey H., Jaramillo A., Dagan D., Griffith L.C., Levitan I.B. (1999). A dynamically regulated 14-3-3, Slob, and Slowpoke potassium channel complex in Drosophila presynaptic nerve terminals. Neuron.

[70] Ichimura T., Isobe T., Okuyama T., Yamauchi T., Fujisawa H. (1987). Brain 14-3-3 protein is an activator protein that activates tryptophan 5-monooxygenase and tyrosine 3-monooxygenase in the presence of Ca^2+^, calmodulin-dependent protein kinase II. FEBS Lett..

[71] Wang J., Lou H., Pedersen C.J., Smith A.D., Perez R.G. (2009). 14-3-3zeta contributes to tyrosine hydroxylase activity in MN9D cells: localization of dopamine regulatory proteins to mitochondria. J. Biol. Chem..

[72] Aitken A. (2006). 14-3-3 proteins: a historic overview. Semin. Cancer Biol..

[73] Toyo-oka K., Wachi T., Hunt R.F., Baraban S.C., Taya S., Ramshaw H., Kaibuchi K., Schwarz Q.P., Lopez A.F., Wynshaw-Boris A. (2014). 14-3-3ε and ζ regulate neurogenesis and differentiation of neuronal progenitor cells in the developing brain. J. Neurosci..

[74] Toyo-oka K., Shionoya A., Gambello M.J., Cardoso C., Leventer R., Ward H.L., Ayala R., Tsai L.H., Dobyns W., Ledbetter D. (2003). 14-3-3epsilon is important for neuronal migration by binding to NUDEL: a molecular explanation for Miller-Dieker syndrome. Nat. Genet..

[75] Taya S., Shinoda T., Tsuboi D., Asaki J., Nagai K., Hikita T., Kuroda S., Kuroda K., Shimizu M., Hirotsune S. (2007). DISC1 regulates the transport of the NUDEL/LIS1/14-3-3epsilon complex through kinesin-1. J. Neurosci..

[76] Jaehne E.J., Ramshaw H., Xu X., Saleh E., Clark S.R., Schubert K.O., Lopez A., Schwarz Q., Baune B.T. (2015). In-vivo administration of clozapine affects behaviour but does not reverse dendritic spine deficits in the 14-3-3ζ KO mouse model of schizophrenia-like disorders. Pharmacol. Biochem. Behav..

[77] Xu X., Jaehne E.J., Greenberg Z., McCarthy P., Saleh E., Parish C.L., Camera D., Heng J., Haas M., Baune B.T. (2015). 14-3-3ζ deficient mice in the BALB/c background display behavioural and anatomical defects associated with neurodevelopmental disorders. Sci. Rep..

[78] Wong A.H., Likhodi O., Trakalo J., Yusuf M., Sinha A., Pato C.N., Pato M.T., Van Tol H.H., Kennedy J.L. (2005). Genetic and post-mortem mRNA analysis of the 14-3-3 genes that encode phosphoserine/threonine-binding regulatory proteins in schizophrenia and bipolar disorder. Schizophr. Res..

[79] Jia Y., Yu X., Zhang B., Yuan Y., Xu Q., Shen Y., Shen Y. (2004). An association study between polymorphisms in three genes of 14-3-3 (tyrosine 3-monooxygenase/tryptophan 5-monooxygenase activation protein) family and paranoid schizophrenia in northern Chinese population. Eur. Psychiatry.

[80] Cheah P.S., Ramshaw H.S., Thomas P.Q., Toyo-Oka K., Xu X., Martin S., Coyle P., Guthridge M.A., Stomski F., van den Buuse M. (2012). Neurodevelopmental and neuropsychiatric behaviour defects arise from 14-3-3ζ deficiency. Mol. Psychiatry.

[81] Fromer M., Pocklington A.J., Kavanagh D.H., Williams H.J., Dwyer S., Gormley P., Georgieva L., Rees E., Palta P., Ruderfer D.M. (2014). De novo mutations in schizophrenia implicate synaptic networks. Nature.

[82] Vawter M.P., Barrett T., Cheadle C., Sokolov B.P., Wood W.H., Donovan D.M., Webster M., Freed W.J., Becker K.G. (2001). Application of cDNA microarrays to examine gene expression differences in schizophrenia. Brain Res. Bull..

[83] English J.A., Pennington K., Dunn M.J., Cotter D.R. (2011). The neuroproteomics of schizophrenia. Biol. Psychiatry.

[84] Potash J.B., Zandi P.P., Willour V.L., Lan T.H., Huo Y., Avramopoulos D., Shugart Y.Y., MacKinnon D.F., Simpson S.G., McMahon F.J. (2003). Suggestive linkage to chromosomal regions 13q31 and 22q12 in families with psychotic bipolar disorder. Am. J. Psychiatry.

[85] Fallin M.D., Lasseter V.K., Avramopoulos D., Nicodemus K.K., Wolyniec P.S., McGrath J.A., Steel G., Nestadt G., Liang K.Y., Huganir R.L. (2005). Bipolar I disorder and schizophrenia: A 440-single-nucleotide polymorphism screen of 64 candidate genes among Ashkenazi Jewish case-parent trios. Am. J. Hum. Genet..

[86] Grover D., Verma R., Goes F.S., Mahon P.L., Gershon E.S., McMahon F.J., Potash J.B., Gershon E.S., McMahon F.J., Potash J.B. (2009). Family-based association of YWHAH in psychotic bipolar disorder. Am. J. Med. Genet. B Neuropsychiatr. Genet..

[87] Pers T.H., Hansen N.T., Lage K., Koefoed P., Dworzynski P., Miller M.L., Flint T.J., Mellerup E., Dam H., Andreassen O.A. (2011). Meta-analysis of heterogeneous data sources for genome-scale identification of risk genes in complex phenotypes. Genet. Epidemiol..

[88] Elashoff M., Higgs B.W., Yolken R.H., Knable M.B., Weis S., Webster M.J., Barci B.M., Torrey E.F. (2007). Meta-analysis of 12 genomic studies in bipolar disorder. J. Mol. Neurosci..

[89] Thapa N., Lee B.H., Kim I.S. (2007). TGFBIp/betaig-h3 protein: A versatile matrix molecule induced by TGF-beta. Int. J. Biochem. Cell Biol..

[90] Billings P.C., Whitbeck J.C., Adams C.S., Abrams W.R., Cohen A.J., Engelsberg B.N., Howard P.S., Rosenbloom J. (2002). The transforming growth factor-beta-inducible matrix protein (beta)ig-h3 interacts with fibronectin. J. Biol. Chem..

[91] Kim J.E., Jeong H.W., Nam J.O., Lee B.H., Choi J.Y., Park R.W., Park J.Y., Kim I.S. (2002). Identification of motifs in the fasciclin domains of the transforming growth factor-beta-induced matrix protein betaig-h3 that interact with the alphavbeta5 integrin. J. Biol. Chem..

[92] Reinboth B., Thomas J., Hanssen E., Gibson M.A. (2006). Beta ig-h3 interacts directly with biglycan and decorin, promotes collagen VI aggregation, and participates in ternary complexing with these macromolecules. J. Biol. Chem..

[93] Lee B.H., Bae J.S., Park R.W., Kim J.E., Park J.Y., Kim I.S. (2006). betaig-h3 triggers signaling pathways mediating adhesion and migration of vascular smooth muscle cells through alphavbeta5 integrin. Exp. Mol. Med..

[94] Thapa N., Kang K.B., Kim I.S. (2005). Beta ig-h3 mediates osteoblast adhesion and inhibits differentiation. Bone.

[95] Yun S.J., Kim M.O., Kim S.O., Park J., Kwon Y.K., Kim I.S., Lee E.H. (2002). Induction of TGF-beta-inducible gene-h3 (betaig-h3) by TGF-beta1 in astrocytes: implications for astrocyte response to brain injury. Brain Res. Mol. Brain Res..

[96] Shi J.D., Kukar T., Wang C.Y., Li Q.Z., Cruz P.E., Davoodi-Semiromi A., Yang P., Gu Y., Lian W., Wu D.H. (2001). Molecular cloning and characterization of a novel mammalian endo-apyrase (LALP1). J. Biol. Chem..

[97] Seo J., Osorio J.S., Schmitt E., Corrêa M.N., Bertoni G., Trevisi E., Loor J.J. (2014). Hepatic purinergic signaling gene network expression and its relationship with inflammation and oxidative stress biomarkers in blood from peripartal dairy cattle. J. Dairy Sci..

[98] Tordella L., Khan S., Hohmeyer A., Banito A., Klotz S., Raguz S., Martin N., Dhamarlingam G., Carroll T., González Meljem J.M. (2016). SWI/SNF regulates a transcriptional program that induces senescence to prevent liver cancer. Genes. Dev..

[99] Gaudet P., Livstone M.S., Lewis S.E., Thomas P.D. (2011). Phylogenetic-based propagation of functional annotations within the Gene Ontology consortium. Brief. Bioinform..

[100] Wang Z., Tseng C.P., Pong R.C., Chen H., McConnell J.D., Navone N., Hsieh J.T. (2002). The mechanism of growth-inhibitory effect of DOC-2/DAB2 in prostate cancer. Characterization of a novel GTPase-activating protein associated with N-terminal domain of DOC-2/DAB2. J. Biol. Chem..

[101] Zhang H., He Y., Dai S., Xu Z., Luo Y., Wan T., Luo D., Jones D., Tang S., Chen H. (2008). AIP1 functions as an endogenous inhibitor of VEGFR2-mediated signaling and inflammatory angiogenesis in mice. J. Clin. Investig..

[102] Zhou H.J., Chen X., Huang Q., Liu R., Zhang H., Wang Y., Jin Y., Liang X., Lu L., Xu Z. (2014). AIP1 mediates vascular endothelial cell growth factor receptor-3-dependent angiogenic and lymphangiogenic responses. Arterioscler. Thromb. Vasc. Biol..

[103] Clarke H.J., Chambers J.E., Liniker E., Marciniak S.J. (2014). Endoplasmic reticulum stress in malignancy. Cancer Cell..

[104] Luo D., He Y., Zhang H., Yu L., Chen H., Xu Z., Tang S., Urano F., Min W. (2008). AIP1 is critical in transducing IRE1-mediated endoplasmic reticulum stress response. J. Biol. Chem..

[105] Zhang H., Zhang H., Lin Y., Li J., Pober J.S., Min W. (2007). RIP1-mediated AIP1 phosphorylation at a 14-3-3-binding site is critical for tumor necrosis factor-induced ASK1-JNK/p38 activation. J. Biol. Chem..

[106] Di Minin G., Bellazzo A., Dal Ferro M., Chiaruttini G., Nuzzo S., Bicciato S., Piazza S., Rami D., Bulla R., Sommaggio R. (2014). Mutant p53 reprograms TNF signaling in cancer cells through interaction with the tumor suppressor DAB2IP. Mol. Cell..

[107] Zhang R., He X., Liu W., Lu M., Hsieh J.T., Min W. (2003). AIP1 mediates TNF-alpha-induced ASK1 activation by facilitating dissociation of ASK1 from its inhibitor 14-3-3. J. Clin. Investig..

[108] Qiao S., Homayouni R. (2015). Dab2IP Regulates Neuronal Positioning, Rap1 Activity and Integrin Signaling in the Developing Cortex. Dev. Neurosci..

[109] Qiao S., Kim S.H., Heck D., Goldowitz D., LeDoux M.S., Homayouni R. (2013). Dab2IP GTPase activating protein regulates dendrite development and synapse number in cerebellum. PLoS ONE.

[110] Moore L.D., Le T., Fan G. (2013). DNA Methylation and Its Basic Function. Neuropsychopharmacol.

[111] Shoichet B.K., Kobilka B.K. (2012). Structure-based drug screening for G-protein-coupled receptors. Trends Pharmacol. Sci..

[112] Denis C., Saulière A., Galandrin S., Sénard J.M., Galés C. (2012). Probing heterotrimeric G protein activation: applications to biased ligands. Curr. Pharm. Des..

[113] Hauser A.S., Attwood M.M., Rask-Andersen M., Schiöth H.B., Gloriam D.E. (2017). Trends in GPCR drug discovery: new agents, targets and indications. Nat. Rev. Drug Discov..

[114] Sriram K., Insel P.A. (2018). G Protein-Coupled Receptors as Targets for Approved Drugs: How Many Targets and How Many Drugs?. Mol. Pharmacol..

[115] Purcell R.H., Hall R.A. (2018). Adhesion G Protein-Coupled Receptors as Drug Targets. Annu. Rev. Pharmacol. Toxicol..

[116] Hamann J., Aust G., Araç D., Engel F.B., Formstone C., Fredriksson R., Hall R.A., Harty B.L., Kirchhoff C., Knapp B. (2015). International Union of Basic and Clinical Pharmacology. XCIV. Adhesion G protein-coupled receptors. Pharmacol. Rev..

[117] Ganesh R.A., Venkataraman K., Sirdeshmukh R. (2020). GPR56: An adhesion GPCR involved in brain development, neurological disorders and cancer. Brain Res..

[118] Langenhan T., Piao X., Monk K.R. (2016). Adhesion G protein-coupled receptors in nervous system development and disease. Nat. Rev. Neurosci..

[119] Duman J.G., Tzeng C.P., Tu Y.K., Munjal T., Schwechter B., Ho T.S., Tolias K.F. (2013). The adhesion-GPCR BAI1 regulates synaptogenesis by controlling the recruitment of the Par3/Tiam1 polarity complex to synaptic sites. J. Neurosci..

[120] Zhu D., Li C., Swanson A.M., Villalba R.M., Guo J., Zhang Z., Matheny S., Murakami T., Stephenson J.R., Daniel S. (2015). BAI1 regulates spatial learning and synaptic plasticity in the hippocampus. J. Clin. Investig..

[121] Stephenson J.R., Paavola K.J., Schaefer S.A., Kaur B., Van Meir E.G., Hall R.A. (2013). Brain-specific angiogenesis inhibitor-1 signaling, regulation, and enrichment in the postsynaptic density. J. Biol. Chem..

[122] Tu Y.K., Duman J.G., Tolias K.F. (2018). The Adhesion-GPCR BAI1 Promotes Excitatory Synaptogenesis by Coordinating Bidirectional Trans-synaptic Signaling. J. Neurosci..

[123] Carr C.M., Rizo J. (2010). At the junction of SNARE and SM protein function. Curr. Opin. Cell Biol..

[124] Hou N., Yang Y., Scott I.C., Lou X. (2017). The Sec domain protein Scfd1 facilitates trafficking of ECM components during chondrogenesis. Dev. Biol..

[125] Nogueira C., Erlmann P., Villeneuve J., Santos A.J., Martínez-Alonso E., Martínez-Menárguez J.Á., Malhotra V. (2014). SLY1 and Syntaxin 18 specify a distinct pathway for procollagen VII export from the endoplasmic reticulum. eLife.

[126] Bando Y., Katayama T., Taniguchi M., Ishibashi T., Matsuo N., Ogawa S., Tohyama M. (2005). RA410/Sly1 suppresses MPP+ and 6-hydroxydopamine-induced cell death in SH-SY5Y cells. Neurobiol. Dis..

[127] Chen Y., Zhou Q., Gu X., Wei Q., Cao B., Liu H., Hou Y., Shang H. (2018). An association study between SCFD1 rs10139154 variant and amyotrophic lateral sclerosis in a Chinese cohort. Amyotroph. Lateral Scler. Front. Degener.

[128] Aoki Y., Manzano R., Lee Y., Dafinca R., Aoki M., Douglas A.G.L., Varela M.A., Sathyaprakash C., Scaber J., Barbagallo P. (2017). C9orf72 and RAB7L1 regulate vesicle trafficking in amyotrophic lateral sclerosis and frontotemporal dementia. Brain.

[129] Jovičić A., Mertens J., Boeynaems S., Bogaert E., Chai N., Yamada S.B., Paul J.W., Sun S., Herdy J.R., Bieri G. (2015). Modifiers of C9orf72 dipeptide repeat toxicity connect nucleocytoplasmic transport defects to FTD/ALS. Nat. Neurosci..

[130] Theuns J., Verstraeten A., Sleegers K., Wauters E., Gijselinck I., Smolders S., Crosiers D., Corsmit E., Elinck E., Sharma M. (2014). GEO-PD Consortium. Global investigation and meta-analysis of the C9orf72 (G4C2)n repeat in Parkinson disease. Neurology.

[131] Conlon E.G., Fagegaltier D., Agius P., Davis-Porada J., Gregory J., Hubbard I., Kang K., Kim D., Phatnani H., Shneider N.A. (2018). Unexpected similarities between C9ORF72 and sporadic forms of ALS/FTD suggest a common disease mechanism. eLife.

[132] Haeusler A.R., Donnelly C.J., Periz G., Simko E.A., Shaw P.G., Kim M.S., Maragakis N.J., Troncoso J.C., Pandey A., Sattler R. (2014). C9orf72 nucleotide repeat structures initiate molecular cascades of disease. Nature.

[133] Muresan V., Ladescu Muresan Z. (2016). Shared Molecular Mechanisms in Alzheimer’s Disease and Amyotrophic Lateral Sclerosis: Neurofilament-Dependent Transport of sAPP, FUS, TDP-43 and SOD1, with Endoplasmic Reticulum-like Tubules. Neurodegener. Dis..

[134] Dardiotis E., Karampinis E., Siokas V., Aloizou A.M., Rikos D., Ralli S., Papadimitriou D., Bogdanos D.P., Hadjigeorgiou G.M. (2019). ERCC6L2 rs591486 polymorphism and risk for amyotrophic lateral sclerosis in Greek population. Neurol. Sci..

[135] Jouroukhin Y., Ostritsky R., Assaf Y., Pelled G., Giladi E., Gozes I. (2013). NAP (davunetide) modifies disease progression in a mouse model of severe neurodegeneration: protection against impairments in axonal transport. Neurobiol. Dis..

[136] Wennerberg K., Rossman K.L., Der C.J. (2005). The Ras superfamily at a glance. J. Cell Sci..

[137] Dawe A.L., Caldwell K.A., Harris P.M., Morris N.R., Caldwell G.A. (2001). Evolutionarily conserved nuclear migration genes required for early embryonic development in Caenorhabditis elegans. Dev. Genes. Evol..

[138] Cockell M.M., Baumer K., Gönczy P. (2004). *lis-1* is required for dynein-dependent cell division processes in *C. elegans embryos*. J. Cell Sci..

[139] Locke C.J., Williams S.N., Schwarz E.M., Caldwell G.A., Caldwell K.A. (2006). Genetic interactions among cortical malformation genes that influence susceptibility to convulsions in *C. elegans*. Brain Res..

[140] Williams S.N., Locke C.J., Braden A.L., Caldwell K.A., Caldwell G.A. (2004). Epileptic-like convulsions associated with LIS-1 in the cytoskeletal control of neurotransmitter signaling in *Caenorhabditis elegans*. Hum. Mol. Genet..

[141] Johnson M., Sharma M., Brocardo M.G., Henderson B.R. (2011). IQGAP1 translocates to the nucleus in early S-phase and contributes to cell cycle progression after DNA replication arrest. Int. J. Biochem. Cell Biol..

[142] Li Z., McNulty D.E., Marler K.J., Lim L., Hall C., Annan R.S., Sacks D.B. (2005). IQGAP1 promotes neurite outgrowth in a phosphorylation-dependent manner. J. Biol. Chem..

[143] Wang Y.N., Wang H., Yamaguchi H., Lee H.J., Lee H.H., Hung M.C. (2010). COPI-mediated retrograde trafficking from the Golgi to the ER regulates EGFR nuclear transport. Biochem. Biophys. Res. Commun..

[144] Hu W., Lin X., Chen K. (2015). Integrated analysis of differential gene expression profiles in hippocampi to identify candidate genes involved in Alzheimer’s disease. Mol. Med. Rep..

[145] Bettayeb K., Hooli B.V., Parrado A.R., Randolph L., Varotsis D., Aryal S., Gresack J., Tanzi R.E., Greengard P., Flajolet M. (2016). Relevance of the COPI complex for Alzheimer’s disease progression in vivo. Proc. Natl. Acad. Sci. USA.

[146] Jain Goyal M., Zhao X., Bozhinova M., Andrade-López K., de Heus C., Schulze-Dramac S., Müller-McNicoll M., Klumperman J., Béthune J. (2020). A paralog-specific role of COPI vesicles in the neuronal differentiation of mouse pluripotent cells. Life Sci. Alliance.

[147] Laemmli U.K. (1970). Cleavage of structural proteins during the assembly of the head of bacteriophage T4. Nature.

[148] Rusanov A.L., Kozhin P.M., Tikhonova O.V., Zgoda V.G., Loginov D.S., Chlastáková A., Selinger M., Sterba J., Grubhoffer L., Luzgina N.G. (2021). Proteome Profiling of PMJ2-R and Primary Peritoneal Macrophages. Int. J. Mol. Sci..

[149] Geer L.Y., Markey S.P., Kowalak J.A., Wagner L., Xu M., Maynard D.M., Yang X., Shi W., Bryant S.H. (2004). Open mass spectrometry search algorithm. J. Proteome Res..

[150] Craig R., Beavis R.C. (2004). TANDEM: matching proteins with tandem mass spectra. Bioinformatics.

[151] Barsnes H., Vaudel M. (2018). SearchGUI: A Highly Adaptable Common Interface for Proteomics Search and de Novo Engines. J. Proteome Res..

[152] Elias J.E., Gygi S.P. (2010). Target-decoy search strategy for mass spectrometry-based proteomics. Methods Mol. Biol..

[153] Vaudel M., Burkhart J.M., Zahedi R.P., Oveland E., Berven F.S., Sickmann A., Martens L., Barsnes H. (2015). PeptideShaker enables reanalysis of MS-derived proteomics data sets. Nat. Biotechnol..

[154] Vaudel M., Breiter D., Beck F., Rahnenführer J., Martens L., Zahedi R.P. (2013). D-score: a search engine independent MD-score. Proteomics.

[155] Taus T., Köcher T., Pichler P., Paschke C., Schmidt A., Henrich C., Mechtler K. (2011). Universal and confident phosphorylation site localization using phosphoRS. J. Proteome Res..

[156] Barsnes H., Vaudel M., Colaert N., Helsens K., Sickmann A., Berven F.S., Martens L. (2011). Compomics-utilities: an open-source Java library for computational proteomics. BMC Bioinform..

[157] Paoletti A.C., Parmely T.J., Tomomori-Sato C., Sato S., Zhu D., Conaway R.C., Conaway J.W., Florens L., Washburn M.P. (2006). Quantitative proteomic analysis of distinct mammalian Mediator complexes using normalized spectral abundance factors. Proc. Natl. Acad. Sci. USA.

[158] McIlwain S., Mathews M., Bereman M.S., Rubel E.W., MacCoss M.J., Noble W.S. (2012). Estimating relative abundances of proteins from shotgun proteomics data. BMC Bioinform..

[159] Degroeve S., Staes A., De Bock P.J., Martens L. (2012). The effect of peptide identification search algorithms on MS2-based label-free protein quantification. OMICS.

[160] Florens L., Carozza M.J., Swanson S.K., Fournier M., Coleman M.K., Workman J.L., Washburn M.P. (2006). Analyzing chromatin remodeling complexes using shotgun proteomics and normalized spectral abundance factors. Methods.

[161] Geraghty N.J., Satapathy S., Kelly M., Cheng F., Lee A., Wilson M.R. (2021). Expanding the family of extracellular chaperones: Identification of human plasma proteins with chaperone activity. Protein Sci..

[162] Rossouw S.C., Bendou H., Blignaut R.J., Bell L., Rigby J., Christoffels A. (2021). Evaluation of Protein Purification Techniques and Effects of Storage Duration on LC-MS/MS Analysis of Archived FFPE Human CRC Tissues. Pathol. Oncol. Res..

[163] Van der Pan K., Kassem S., Khatri I., de Ru Arnoud H., Janssen George M.C., Tjokrodirijo Rayman T.N., al Makindji F., Stavrakaki E., de Jager Anniek L., Naber Brigitta A.E. (2022). Quantitative proteomics of small numbers of closely-related cells: Selection of the optimal method for a clinical setting. Front. Med..

